# Selective Attention Modulates the Direction of Audio-Visual Temporal Recalibration

**DOI:** 10.1371/journal.pone.0099311

**Published:** 2014-07-08

**Authors:** Nara Ikumi, Salvador Soto-Faraco

**Affiliations:** 1 Departament de Tecnologies de la Informació i les Comunicacions, Universitat Pompeu Fabra, Barcelona, Spain; 2 Institució Catalana de Recerca i Estudis Avançats (ICREA), Barcelona, Spain; University of California, Davis, United States of America

## Abstract

Temporal recalibration of cross-modal synchrony has been proposed as a mechanism to compensate for timing differences between sensory modalities. However, far from the rich complexity of everyday life sensory environments, most studies to date have examined recalibration on isolated cross-modal pairings. Here, we hypothesize that selective attention might provide an effective filter to help resolve which stimuli are selected when multiple events compete for recalibration. We addressed this question by testing audio-visual recalibration following an adaptation phase where two opposing audio-visual asynchronies were present. The direction of voluntary visual attention, and therefore to one of the two possible asynchronies (flash leading or flash lagging), was manipulated using colour as a selection criterion. We found a shift in the point of subjective audio-visual simultaneity as a function of whether the observer had focused attention to audio-then-flash or to flash-then-audio groupings during the adaptation phase. A baseline adaptation condition revealed that this effect of endogenous attention was only effective toward the lagging flash. This hints at the role of exogenous capture and/or additional endogenous effects producing an asymmetry toward the leading flash. We conclude that selective attention helps promote selected audio-visual pairings to be combined and subsequently adjusted in time but, stimulus organization exerts a strong impact on recalibration. We tentatively hypothesize that the resolution of recalibration in complex scenarios involves the orchestration of top-down selection mechanisms and stimulus-driven processes.

## Introduction

Coherent perception of multisensory events requires the adjustment of temporal discrepancies in physical transmission and neural processing times between sensory modalities [1]–[4]. This is so because, although temporal proximity between the stimuli is often claimed to be necessary for binding multisensory events [5], perceptual simultaneity is not necessarily correlated with physical synchrony. For example, the perception of audio-visual simultaneity is often maximal when light appears slightly ahead of sound [3], [6]. Hence, information processing in the brain must be flexible to re-align in time the information received through different senses in order to form coherent cross-modal representations. Several mechanisms, not necessarily incompatible with each other, might be involved in adjusting these temporal discrepancies among the senses. Some authors propose the existence of a temporal window within which simultaneity of cross-modal events is perceived despite small temporal disparities, with estimates ranging from 25–50 ms [7]–[9] to 150–250 ms, for complex stimuli such as music or speech [10]–[12]. Another putative mechanism for maintaining temporal coherence across modalities is temporal ventriloquism, whereby a sound may attract the perceived onset of a temporally close visual event [13]–[15]. In addition, several studies suggest an intriguing mechanism of recalibration based on perceptual adaptation to cross-modal asynchrony [16], [17]. Typically, in the recalibration paradigm, the subjective point of simultaneity for cross-modal pairs of stimuli shifts after a short adaptation phase where this cross-modal pairing is presented repeatedly at a constant asynchrony. The finding of temporal recalibration is interesting because it suggests a remarkable short-term plasticity that allows the perceptual system to adjust to the changing conditions of the environment, such as the arrival times of sound and light as distance between the observer and the multisensory object varies [18].

What we address here is the role of selective attention in this process. The reason is that, in contrast with the simplified laboratory conditions typically used to test recalibration (where only the two relevant stimuli are usually present at one trial), in everyday life our perceptual system must deal with a multitude of sensory inputs, often occurring close or at overlapping times. Thus one might wonder if maintaining a single estimate of synchrony [10], [16], [19]–[23] is useful or even possible in these sensory complex conditions. Here we address the hypothesis that attention helps the selection of which stimuli pair is recalibrated, out of all the information received. Past studies suggest that one potential filter for temporal recalibration might be cross-modal congruence in terms of stimulus-based features such as spatial location or prior learned associations. Indeed, Yarrow, Roseboom, & Arnold (2011) [24] demonstrated that presentation of a stream of audio-visual temporal ambiguous stimuli, at different spatial locations, induced a spatially specific recalibration effect. The role of other stimulus properties is more controversial. For example Heron, Roach, Hanson, McGraw, & Whitaker (2012) [25] demonstrated adaptation to two opposing temporal asynchronies when flashes and tones are separated in space but not if their pairing was only based on contextual associations (high and low pitch sounds coupled to a specific visual spatial frequencies). In a recent study, Roseboom, Kawabe, & Nishida (2013) [26] demonstrated recalibration of opposing asynchronies of spatially overlapping audio-visual events based on prior associations of physical properties of the stimuli (vertical or horizontal gabor patch coupled to a specific pitch sound) and gender congruency (faces and voices) strengthening previous work [27]. These results speak to the influence of stimulus-based factors on the ability to recalibrate in time across sensory modalities. Here, instead, we addressed whether recalibration may depend also on endogenous attentional selection. That is, do attended stimuli have preferential access to the recalibration mechanism? We hypothesize that endogenous attention might be a candidate to help the system filter which cross-modal pairings would be adjusted in time when multiple sensory events are present, and therefore compete for recalibration.

Unlike previous studies examining the effect of attention on the magnitude of recalibration [16], [28], we investigated the role of endogenous attention driving the selection of which stimuli pair should be adjusted in time, examining the direction of the recalibration after-effects. In particular, we addressed whether deploying attention towards one of two competing audiovisually asynchronous events segregated by colour, can shift the point of subjective simultaneity (PSS) in the direction of the attended asynchrony and away from the unattended one. The experimental task was adapted from Fujisaki et al's.,(2004) [16] seminal demonstration of recalibration but, instead of exposing participants to one isolated audio-visual asynchrony (flash-tone or tone-flash), we presented them with two competing asynchronies using triplet sequences of flash-tone-flash. During the adaptation phase, participants' attention was directed to a visual feature of either the flash preceding (attend leading flash condition) or the flash trailing (attend lagging flash condition) the tone in separate sessions. We hypothesize that, if selection by endogenous attention determines the direction of recalibration, then physically identical exposure conditions would lead to radically different after-effects only by changes in the observer's focus of attention. Alternatively, if recalibration is an automatic mechanism, only depending on stimulus-based features, then the exposure to the two competing asynchronies should resolve in the same recalibration outcome no matter what events are endogenously attended. Our findings in Experiment 1 showed that selective attention during adaptation, modulates subjective simultaneity between vision and audition. However, an overall recalibration asymmetry towards vision leading asynchronies was found when contrasting PSS's before and after adaptation. A follow up, Experiment 2 confirmed this asymmetry and suggested that both, stimulus-driven and top-down process compete to determine which stimulus pairs should be adjusted in time.

## Methods

### Ethics Statement

All procedures had been previously approved by the local ethical board (CEIC Parc de Mar).

### Subjects

Thirty-nine paid volunteers who were naive about the purpose of the experiments plus one of the authors (N.I.), between ages of 18 and 38 participated in the experiment (18 in Experiment 1, and 22 in Experiment 2). All had normal or corrected-to-normal vision and hearing. Informed consent was obtained in written.

### Apparatus

We ran the experiments on a PC using Psychotoolbox toolbox on Matlab R2010b. The participant sat at the distance of 50 cm from the monitor (‘PHILLIPS109B’, 85 hz, 800×600 pixels), with their head resting on a chinrest, in a quiet dark room. The auditory stimuli were presented through headphones (Sennheiser PC 161). Accurate timing and synchronization of auditory and visual stimuli was achieved using a Blackbox Toolkit (Accuracy of <1 ms; Cambridge Research Systems). We applied gamma correction to our monitor to determine luminance values using a luminometer (Minolta LS-100).

### Stimuli

The visual stimulus was a gabor ring (outer diameter, 5.0°; inner, 2.5°) with vertical gratings (spatial frequency = 0.1 cycles per pixel; phase = 0.25 cycles; sigma = 20 pixels), that flashed for one monitor frame (11.8 ms) at the centre of a black square pedestal (11.6°, 0.8 cd/m^2^) on white background. A fixation marker was presented at the square's centre. The auditory stimulus was a tone (1.8 kHz, 70 [A]dB SPL) lasting for 10 ms with a 2.5 ms raised-cosine ramp at the onset and offset.

### Procedure

**Figure 1:**
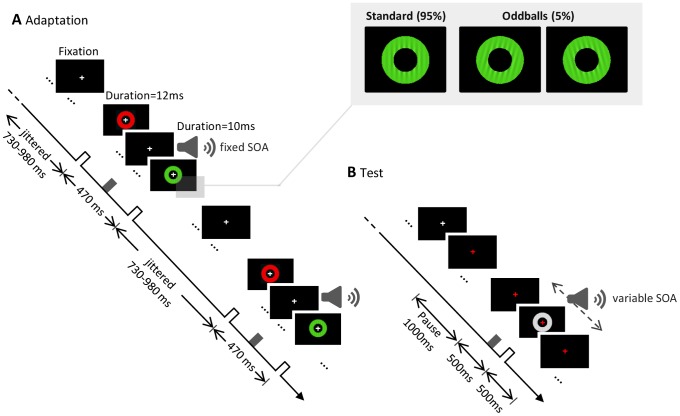
A) Schematic representation of a fragment of the adaptation phase in Experiments 1 and 2. The sequence was composed by a flash, a tone and a flash (in the example, RED appears leading the tone and GREEN lagging the tone). The tone was displayed at the perceptual temporal middle between the flashes (SOA adjusted individually and fixed during the adaptation phase). The inset depicts an example of one standard flash (95%) and the corresponding two possible oddball target stimuli (5%) presented during adaptation and re-adaptation phase in Experiment 1 and 2. B) Example of a test trial in Experiments 1 and 2. The fixation cross changed from white to red and after a 1000 ms pause, the test trial (duration = 1000 ms) started. After 500 ms one GREY flash was presented in the centre of the screen and one tone was displayed at one of nine asynchronies ranging from −353 to +353 ms with respect to the flash. Participants responded whether the GREY flash and the tone were simultaneous or not. The fixation turned back to grey and a new re-adaptation sequence started. (The white background surrounding the black pedestal was not included in the figure).

A session started with a 3 minute initial adaptation phase, immediately followed by a test phase that consisted of a simultaneity judgment (SJ) task on audio-visual pairs. Each test trial was preceded by 8 s re-adaptation sequences. Short re-adaptations are included in order to maintain the recalibration after-effects when new temporal information is provided during the postest measurement [29]. During the adaptation phase and the re-adaptations an audio-visual stream (consisting in flash-tone-flash triplet stimuli, with fixed 470 ms flash to flash onset) was presented. According to a recent study [30], a tone displayed physically in the middle of two flashes is not perceived as such, as sensory systems seems to have a preference for binding audio-visual information when vision leads audition. In order to ensure a balanced competition of audio-visual binding, the exact temporal position of the tone was adjusted prior to the experiment for each participant using a temporal bisection task with an adaptive procedure to find the perceptual middle point between the flashes (see further details in Supporting Information S1). A jitter between 730–980 ms was introduced between triplet to triplet interval during the adaptation and subsequent re-adaptations. One second before each test trial the fixation cross changed from white to red as a warning signal, then a single flash-tone pair was presented amongst 9 possible audio-visual onset asynchronies (SOA) chosen at random and equiprobably (+/−353, +/−235, +/−118, +/−59, 0 ms; negative denotes tone before flash). In these trials, participants judged whether the stimuli were simultaneous or not, by pressing one of two designated keys, in an unspeeded manner. The colour of the ring in the test trials was grey, and isoluminant with the coloured rings in the adaptation phase (see below). During the adaptation phase (and re-adaptations) the visual stimuli (rings) preceding and trailing the tone were coloured consistently in green and red (or vice-versa, colour order counterbalanced across participants). Although it is the first study to demonstrate generalization in chromaticity of visual stimulus, the generalization of the after-effects between black and white colour stimuli in adapted trials and different contrasts polarities in test trials was previously tested by Fujisaki et al., (2004) [16] as well as several generalizations of other stimulus properties within a sensory modality. Prior to the experimental session, the red, green and grey colours were adjusted for isoluminance (task adopted from Cavanagh, MacLeod, & Anstis (1987) [31], see further details in Supporting Information S1) for each subject individually to avoid exogenous orienting capture by luminance differences (during the initial piloting (n = 5) we found that the green ring, with higher luminance contrast than the red one, drove the direction of recalibration independently of the direction of voluntary attention). The colour feature was used as a selection criterion to instruct participants to orient attention to the rings leading or the rings lagging the tone [Fig. 1]. To this purpose, participants were asked to detect deviations from vertical (+/−15°) of the grating on the red (or green, counterbalanced) ring. Both the ring preceding and the ring trailing the sound could bear an oddball (5% probability), but participants were instructed to detect (as quickly as they could, but accurately) only the targets appearing in the attended colour, and ignore the others. The choice of colour cues was to avoid giving explicit information about the temporal position of the flash attended respect to the tone, which we believe could otherwise have lead to potential bias in our paradigm.

**Figure 2:**
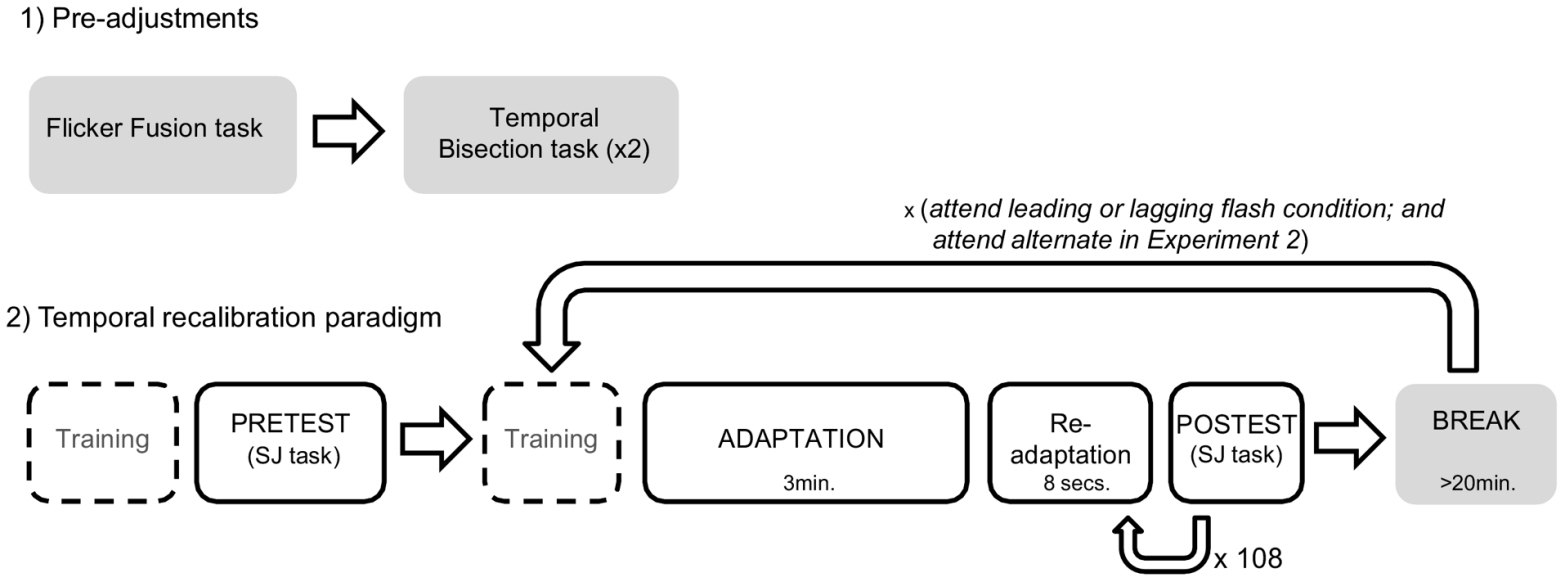
Timeline of the experimental procedure in Experiment 1 and 2. Prior to the temporal recalibration paradigm, participants were asked to perform two tasks. The first of them consisted in matching the red, green and grey stimulus luminance (flicker fusion paradigm). In the latter, the temporal asynchrony of the tone between the two flanking flashes was adjusted (temporal bisection task). After implementing these adjustments in the setting for each participant, a pretest was conducted (simultaneity judgment task). The temporal recalibration paradigm consisted in a 3 minutes adaptation (oddball detection task) followed by a short re-adaptation (oddball detection task) preceding each of the postests (simultaneity judgment task). At the end of the session, 9 different SOAs were tested 12 times each (108 trials). After a break of at least 20 minutes, a new adaptation for another experimental condition was performed. Before the pretest and each adaptation condition, participants ran a training session to ensure the correct understanding of each task.

In a first experiment, each participant performed two 30 minutes sessions with a minimum break of 20 minutes between sessions. Participants were asked to leave the testing room and move around during the break period. The experimenter encouraged conversation during the break to remove possible storage of previous block recalibration after-effect [29]. In each session they adapted to exactly the same physical stimuli, but attended to one of the two colours, which coincided with either the flash preceding (attend leading flash condition) or to the flash trailing the tone (attend lagging flash condition). The temporal position of the attended colour was fixed within a session, but alternated between sessions (order counterbalanced across participants). A session consisted of 108 test trials divided into 12 blocks, with each block containing one instance of the 9 possible SOAs. Thus we obtained 12 observations per test SOA and adaptation condition from each participant. Prior to the adaptation phase, participants ran a pretest, in which they had to perform a SJ task on the 9 audio-visual asynchronies used in the test-phase. Participants familiarized with the different tasks during 2–3 minutes, prior to the pretest and adaptation (see time line of the overall experimental procedure in Fig. 2). There was no feedback during the experiment.

### Data analyses

We determined the proportion of simultaneity responses as a function of test SOAs for the pretest and each attention condition. The data were fitted to a truncated Gaussian function by using the maximum likelihood estimation method (see [32] for further information and Fig. S1 for individual representative fits in the Supporting Information). The free parameters were the mean, denoting the point of subjective simultaneity (PSS), the standard deviation (denoting temporal resolution) and the amplitude of the Gaussian function. Test trials following an attended stream containing oddballs (tilted gratings) during re-adaptations were not included in the analysis. These particular test trials were re-inserted among the remaining test trials of the corresponding block and tested again. Participants whose data had a goodness of fit (R^2^) to the fitted function below 0.5 in one of the conditions (n = 4 in Experiment 1, n = 3 in Experiment 2) were discarded from the analyses (following [33]). We calculated each PSS value in each of the test phases and the pretest. The recalibration magnitude (ΔPSS) was obtained by subtracting PSS values in the test phase following attend leading flash condition, from PSS values after adaptation in the attend lagging flash condition. We were interested in PSS differences in the test phase as a function of which ring (first, leading the tone, or second, trailing the tone) had been attended during adaptation. We applied a Wilcoxon signed rank test of related samples with the IBM SPSS Statistics 19 to assess whether the inter-participants mean differed (level of significance set at 0.05). P-values (p) and z-scores (z) are reported for each of the condition comparisons.

## Experiment 1

### Results

**Figure 3:**
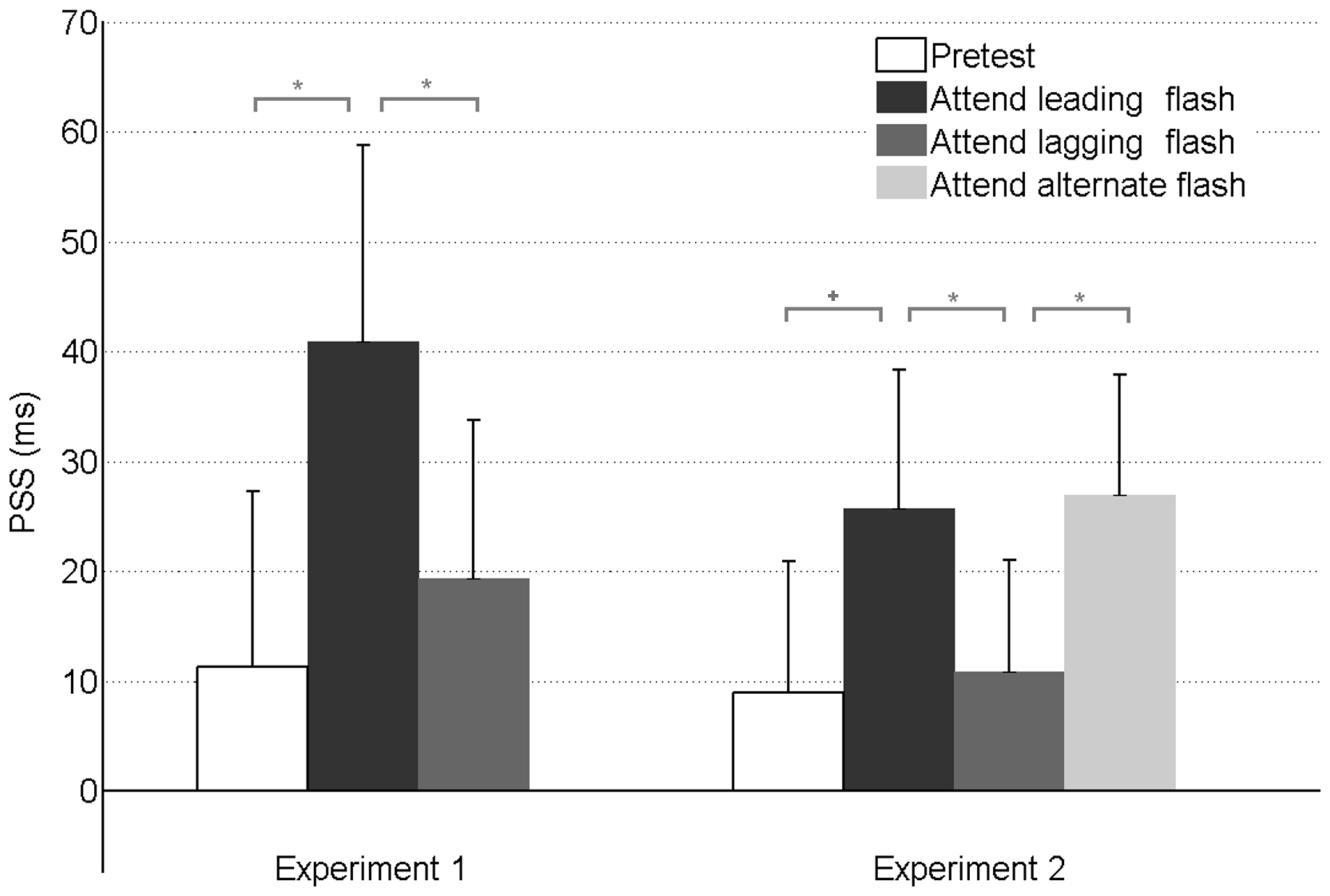
Average PSS values for each condition in Experiments 1 and 2. Average PSS were obtained from the individual means of each participant/condition. Positive PSS values indicate that the flash had to be presented before the tone in order to perceive sound and vision events as simultaneous. The asterisks denote significant differences between the PSS, the cross denotes a marginally significant difference (p = 0.077) and the error bars denote the SEM.

The PSS in the attend leading compared to the attend lagging flash conditions showed a significant shift in the direction of the attended visual stimulus (p = 0.03, z = −2.66. See Table S1 and S2 for a detailed description of the results). In particular, to perceive audio-visual simultaneity, the visual stimulus had to be presented 22 ms (ΔPSS) earlier after adaptation attending the leading flash than after adaptation attending the lagging flash [Fig. 3; and Fig. S2 for individual PSS values]. Additionally, we examined the PSS shift in both test phase conditions compared to pretest, prior to the adaptation to any asynchrony [Fig. S3A]. The results indicated that in the attend leading flash condition, the PSS after adaptation shifted towards the direction of the attended flash (p = 0.016, z = −2.417) relative to the pretest PSS. Instead, no differences were found in the attend lagging flash condition when compared to the pretest PSS (p = 0.397, z = −0.847). Temporal resolution (denoted by standard deviation) of the psychophysical curves was not different between conditions (see Table S1). Proportion of oddball detection for the visual attention task was 59% and 64% for the attend leading and attend lagging flash conditions respectively, not significantly different (p = 0.279, z = −1.083. See Table S3, S4 and S5).

### Discussion

The recalibration magnitude as well as the PSS obtained in the pretest are well in line with previous reports using a SJ task, in which perception of simultaneity tolerates greater vision leading auditory asynchronies [3], [6], [8], [33]–[35]. Remarkably, the present data demonstrate that, under conditions of multiple possible pairings in time, selective attention to particular stimuli during the adaptation can be effective to induce a PSS shift (attend leading vs. attend lagging flash). Note that, in the adaptation phase, the physical stimuli were physically equivalent in both conditions, with the only difference being the particular visual element in the sequence on which participants focused attention. This result does not only imply the role of voluntary attention in cross-modal temporal adaptation, but in addition runs against claims that multisensory integration is an automatic phenomenon that occurs outside the scope of attention (see [36], [37] for a review).

One interesting aspect is that, despite the differential effect induced by voluntary attention between the adapted conditions (attend leading flash or lagging flash), both adaptation conditions led to a positive PSS shift compared to the pretest measurement of perceived simultaneity. That is, compared to the pretest PSS, vision always had to lead audition for simultaneity to be perceived in either post-test, albeit more so in the attend-leading flash (41 ms, p = 0.016, z = −2.417) compared to attend-lagging (19 ms, n.s.). This could be interpreted as endogenous attention only having a significant impact on adaptation in one particular direction of asynchrony. The reason behind this is, however, unclear. Another possible account for this lack of complete symmetry around the pretest PSS value is that the strong saliency of the flash preceding the sound, leading to some automatic capture of exogenous attention common to both conditions, having an effect on top of the endogenous manipulation. For this reason, the presence of two competing stimuli might not necessarily cancel away, given the impact of stimulus-driven factors such as saliency.

Some evidence of the interplay between bottom-up and top-down processes driving the temporal recalibration mechanism was reported in a study addressing the role of attention on the magnitude, instead of the direction, of recalibration [28]. An increase in the recalibration magnitude was found when observers explicitly directed attention to the temporal relation between the stimuli pair during adaptation, compared to some other stimulus feature. Interestingly, in line with our suggestion here, diverting attention away to the stimuli pair did not completely abolish the recalibration after-effects we believe are caused by stimulus-driven factors. Although we cannot rule out the possibility that this asymmetry arises from specific effects of endogenous attention, it is the stimulus properties by itself during adaptation that most probably are leading to this recalibration outcome independently of any voluntary attention. For this reason, the pretest value might not be an appropriate baseline for our attentional effects, because it fails to incorporate possible stimulus-driven factors that might be present during the adaptation phase. In order to test this possibility, we designed a new experiment including a replication of the two attention conditions in Experiment 1 plus an extra adaptation condition (attend to alternate flash) including an attention engagement task of equivalent difficulty, but alternating trial to trial the asynchrony direction of the attended visual event relative to the sound.

## Experiment 2

Like in Experiment 1, participants were asked to search for tilted visual gratings in one colour (red or green) and ignore visual events in the other colour. Two of the conditions replicated the manipulation of Experiment 1 (colours bear a constant place in the temporal sequence, so that participants systematically attended to the first or second flash). A third, additional adaptation condition (attend to alternate flash) was included where ring colour alternated in time with respect to the sound from trial to trial (so that participants attended, in an alternate and consistent fashion, to the leading and lagging visual events) during adaptation.

### Results

We analyzed the PSS obtained after the attend leading and attend lagging flash conditions (as in Experiment 1), as well as in the new attend alternate condition (see Table S1 and S2 for detailed information). The PSS shifted in the direction of attention when comparing attend leading and attend lagging conditions (ΔPSS = 14.8 ms p = 0.03, z = −2.173), thus replicating the modulation by endogenous attention reported in Experiment 1. Also, like in Experiment 1, only the attend leading flash PSS (albeit marginally significant, p = 0.077, z = −1.771), but not the attend lagging flash PSS (p = 0.872, z = −0.161), was different relative to the pretest PSS [Fig. S3A]. So far the data in Experiment 2 is perfectly comparable to those of Experiment 1. However, the important test is the comparison of the PSS after the attend leading and lagging flash adapting conditions with the PSS of the new attend alternate flash condition. In this case, the PSS of the attend lagging flash condition shifted in the direction of voluntary attention (p = 0.018, z = −2.374); However, no shift was observed for the attend leading flash condition compared to the attend alternate one (p = 0.809, z = −0.241) [Fig. 3 and Fig. S3B, S4]. Again, neither temporal precision nor the proportion of oddball detection in the attended conditions revealed any significant differences. Proportion of detection were 53%, 66% and 56% for the attend leading, attend lagging, and attend alternate condition respectively (see Table S3, S4 and S5 for a detailed report).

The results from this experiment confirmed the findings of Expermient 1 and, in extension, they support the idea that the flash preceding the tone might be naturally more salient than the flash lagging. According to this hypothesis then, capture from first flash must have been equal in all adaptation conditions including the baseline (attend leading, lagging and alternate), only when endogenous attention counters this capture (attend lagging), some effect could be observed.

## General Discussion

The straightforward finding to emerge from this study is that, in two experiments, cross-modal synchrony judgments shifted as a function of how endogenous attention was deployed during prior adaptation. In our study, the focus of voluntary attention was directed to one out of two opposite asynchronies during adaptation, one where the flash lead a tone, and one in which a flash followed the same tone. Thus, in order to perceive audio-visual events simultaneous after exposure to two competing asynchronies, when participants attended the flash preceding the tone, vision had to be presented earlier than when participants directed their attention to the flash lagging the tone. We would like to emphasize that in this study adaptation streams were physically identical in both attention conditions, and therefore only the direction of endogenous attention can explain this difference. We also found an unexpected recalibration asymmetry towards vision leading asynchronies when comparing the post-adaptation PSS in both conditions with a pretest baseline (i.e., PSS measured prior to adaptation). We attempted to find the origin of this recalibration asymmetry by using an attend-alternate baseline, which provided us with a comparable post-adaptation PSS baseline in terms of the other adaptation conditions like possible attention demands and training (Experiment 2). Participants were required to deploy attention alternatively to visual leading and lagging events within the adaptation phase, under otherwise similar temporal structure of the stimulus sequences (and therefore, under the same effects due to stimulus-driven capture of the first flash). In Experiment 2 we replicated the same voluntary attention effects found in Experiment 1 when comparing attend-lead vs. attend-lagging flash adaptation, confirming an influence of endogenous attention. In addition, consistent with a putative role of stimulus driven effects of the leading flash, the PSS in the attend leading flash condition was equivalent to the PSS for the attend-aternate baseline, whereas the PSS in the attend-lagging flash shifted in the direction of attention. Thus, it appears that exogenous effects due to first flash produced some adaptation through bottom-up process, which could be only countered when endogenous attention pulled adaptation in the opposite direction. We therefore speculate that only when physical properties of the stimuli are not sufficient to drive participants' attention, selective attention can shift the direction of temporal recalibration. However, another possible interpretation for this asymmetry is that endogenous attention has intrinsically stronger effects to drive adaptation in the flash-lagging direction than in the flash-leading direction. Although this question remains open for further research, our preferred interpretation is the former because it is in line with recent findings of recalibration favouring visual leading auditory temporal adjustments, suggesting a more general experience-based process (light usually arrives before the sound at distances >10 meters) [38], [39]. What is clear is that the present results demonstrate, through a typical temporal recalibration paradigm, that when multiple events compete, not only physical stimulus properties already manipulated in previous studies, but also selective attention to the flash lagging the tone, can modulate the direction of recalibration adjustment of sensory modalities in time.

Prior literature addressing the question of how cross-modal timings are adjusted when multiple events are presented during adaptation has investigated the effects of recalibration driven by natural associations such as spatial location [24], [25] or learned, arbitrary ones including associations arising from specific physical properties of the stimuli [25]–[27]. Interestingly, our findings suggest that purely top-down selection towards the flash lagging a tone, can play a role in temporal adjustment and modulate which stimuli should be adjusted in time. This attentional filter can become particularly useful when stimulus organization and spatial location are not sufficient for the system to determine what has to be adjusted in time in complex, multisensory scenes.

Given that in our task, participants were asked to attend to a visual feature but not to sound, one might wonder how these findings relate to the idea of cross-modal prior entry [40], [41], whereby the processing of the attended modality (or location) might be speeded up with respect to the unattended ones [40]–[42]. In fact, Fujisaki et al., in 2004 [16] addressed the question whether temporal recalibration after-effects could be explained by prior entry, by asking participants to attend to one of the modalities during the test trials. Although Fujisaki et al., found PSS shifts according to prior entry, this effect failed to modulate the magnitude of the recalibration after-effect. In our paradigm attention was directed towards one of two visual stimuli (flash leading tone vs. flash lagging tone) during the adaptation phase. If any modality-general prior entry effect would have occurred, should have affected both visual events thus preventing our specific PSS shifts between conditions to be explained by cross-modal prior entry [16].

Relating to our findings, two recent studies [43], [44] have addressed the role of attention in the detection of cross-modal synchrony in cluttered dynamic environments. Both studies clearly suggest that detecting cross-modal coincidence in time depends on the temporal rate of stimulus presentation, and that audio-visual facilitation fails to happen in an automatic fashion (without voluntary attention) at presentation rates above 2–3 Hz. Thus, this would be another example in which just stimulus-driven properties were not sufficient to segregate cross-modal events in dynamic streams. In our study, we used a presentation rate in which cross-modal events cannot be matched easily just by stimulus properties (2.1 Hz visual presentation), thus perhaps allowing voluntary attention to play a role in the selection of competing audio-visual stimuli.

## Conclusions

This study demonstrates that endogenous selective attention can modulate which particular cross-modal events should be recalibrated in time under conditions where several competing cross-modal asynchronies are present. Our finding helps to provide a more complete picture of temporal recalibration in complex multisensory scenarios, in addition to prior results demonstrating the influence of stimulus-based features. According to the present evidence, we advance the hypothesis that the process of recalibrating cross-modal simultaneity in complex contexts is driven by the interplay between bottom-up (stimulus driven) and top-down (endogenous selection) processes in order to filter out information and coherently organize the time of the events.

## Supporting Information

Figure S1 **Representative individual psychometric functions for** **Experiment 1** **and** **2** **.**
(TIF)

Figure S2 **Individual PSS mean values for each condition in** **Experiment 1** **.**
(TIF)

Figure S3 A. PSS shift differences between conditions with respect to the pretest in Experiment 1 and Experiment 2. PSS shifts were obtained from the difference between mean PSS values for the attend leading and attend lagging flash condition and each attended condition with respect to the pretest. B. PSS shift differences between conditions with respect to attend alternate condition in Experiment 2. PSS shifts were obtained from the difference between mean PSS values for the attend leading and attend lagging flash condition and each of the attended condition with respect to a baseline equated for task demands (attend alternate). Positive PSS values indicate that the tested flash had to be presented before the tone to perceive audiovisual events simultaneous. The asterisks denote significant differences of the PSS, the cross denotes a marginally significant difference (p = 0.078) and the error bars denote the SEM calculated from the difference between conditions.
(TIF)

Figure S4 **Individual PSS mean values for each condition in** **Experiment 2** **.**
(TIF)

Table S1 **PSS, Sigma and R^2^ mean estimates for each condition in** **Experiment 1** **and** **2** **.**
(DOC)

Table S2 **Reported Wilcoxon signed Rank test values for related pair samples conditions: PSS in** **Experiment 1** **and** **2** **.**
(DOC)

Table S3 **Mean proportion of oddball detection for each condition in** **Experiment 1** **and** **2** **.**
(DOC)

Table S4 **Reported Wilcoxon signed Rank test values for related pair samples conditions: Proportion of oddball detection in** **Experiment 1** **and** **2** **.**
(DOC)

Table S5 **Proportion of oddball detection for each participant in** **Experiment 1** **and** **2** **.**
(DOC)

Supporting Information S1 (DOC)
