# Supplementary figures and images for: Selective Attention Modulates the Direction of Audio-Visual Temporal Recalibration
Source: PLoS One. 2014 Jul 8;9(7):e99311. doi: 10.1371/journal.pone.0099311 (PMC4086723; doi:10.1371/journal.pone.0099311)

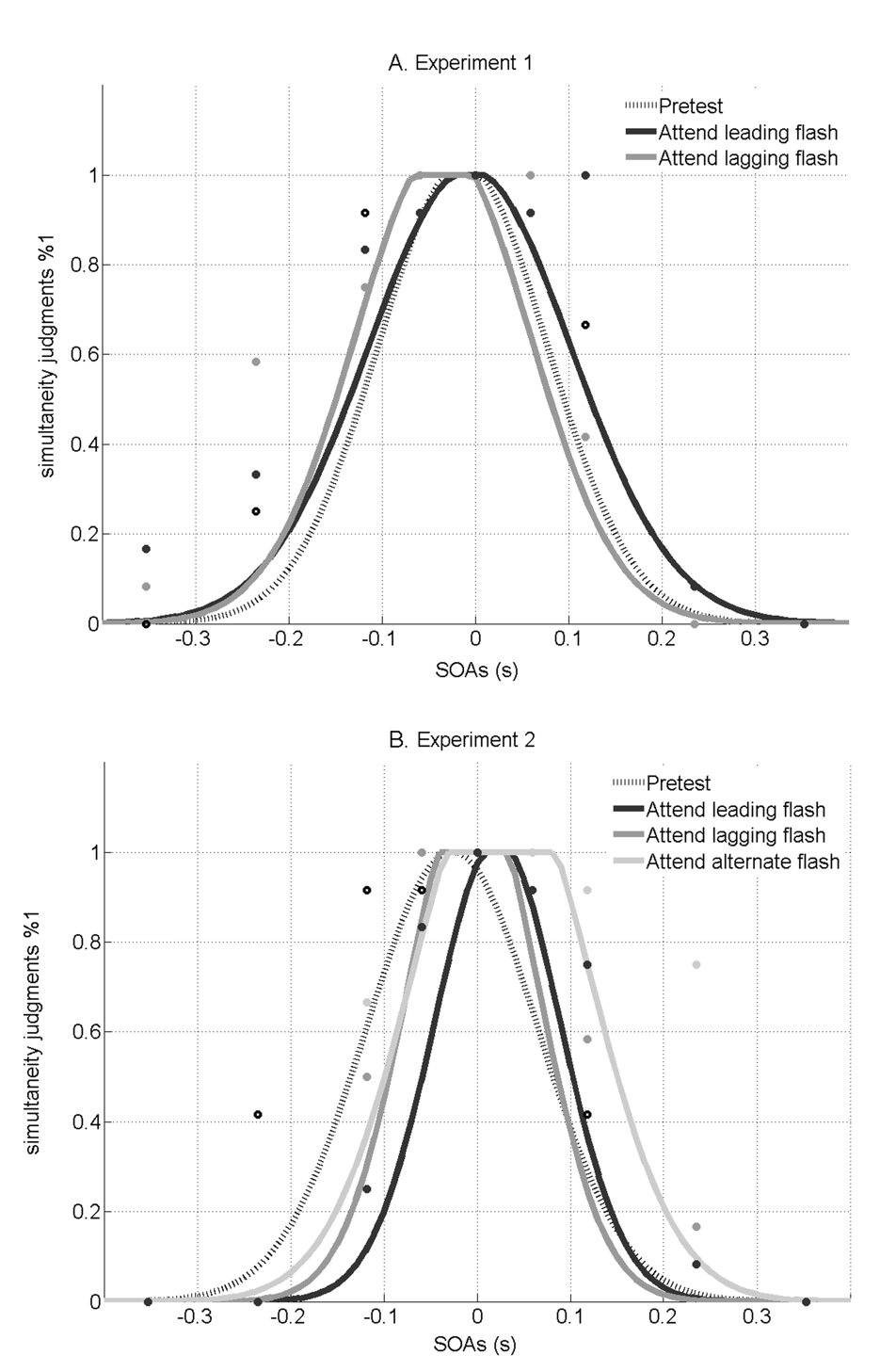

Supplement: Figure S1 — Representative individual psychometric functions for Experiment 1 and 2 . (TIF) [file pone.0099311.s001.tif]

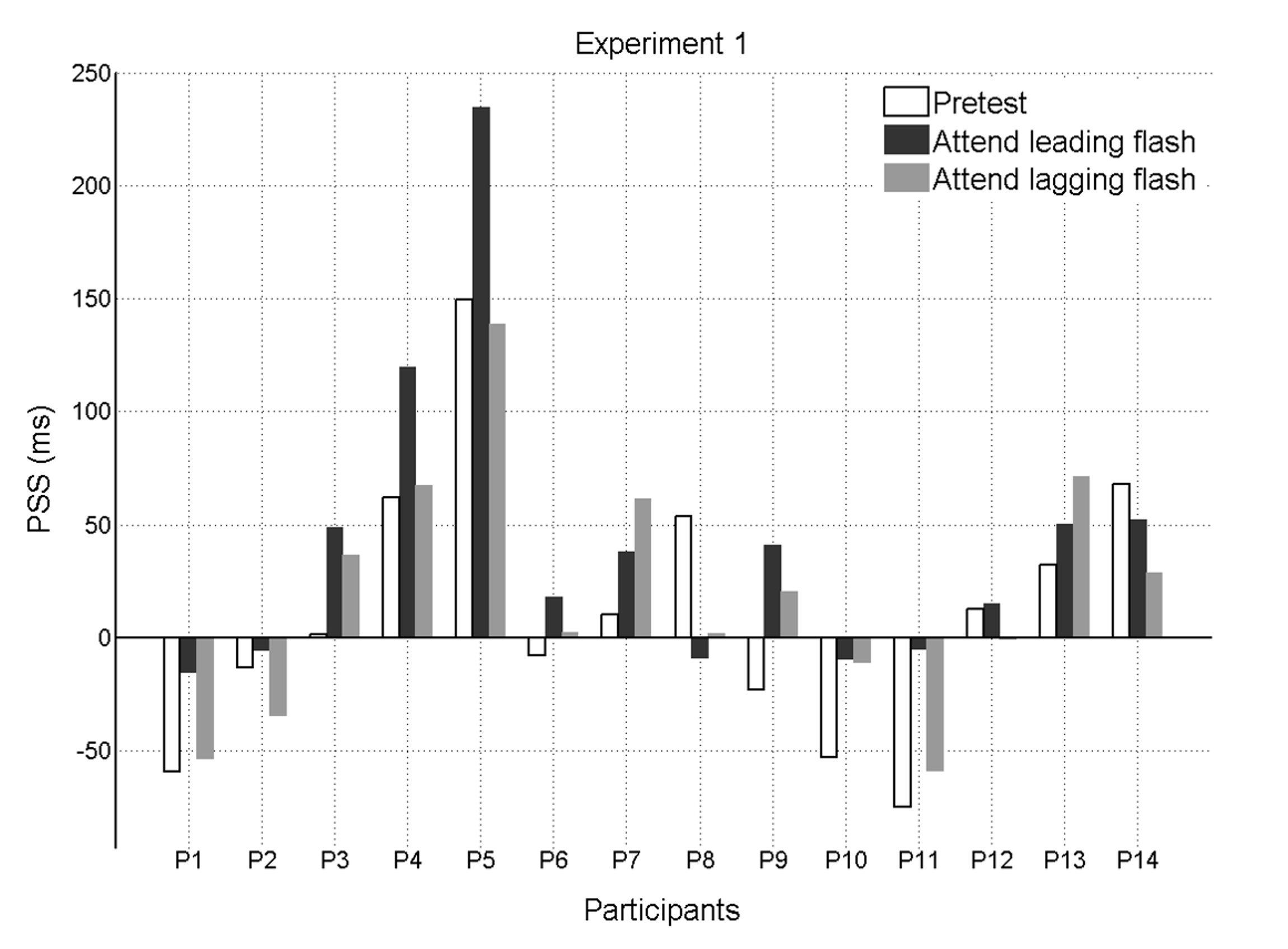

Supplement: Figure S2 — Individual PSS mean values for each condition in Experiment 1 . (TIF) [file pone.0099311.s002.tif]

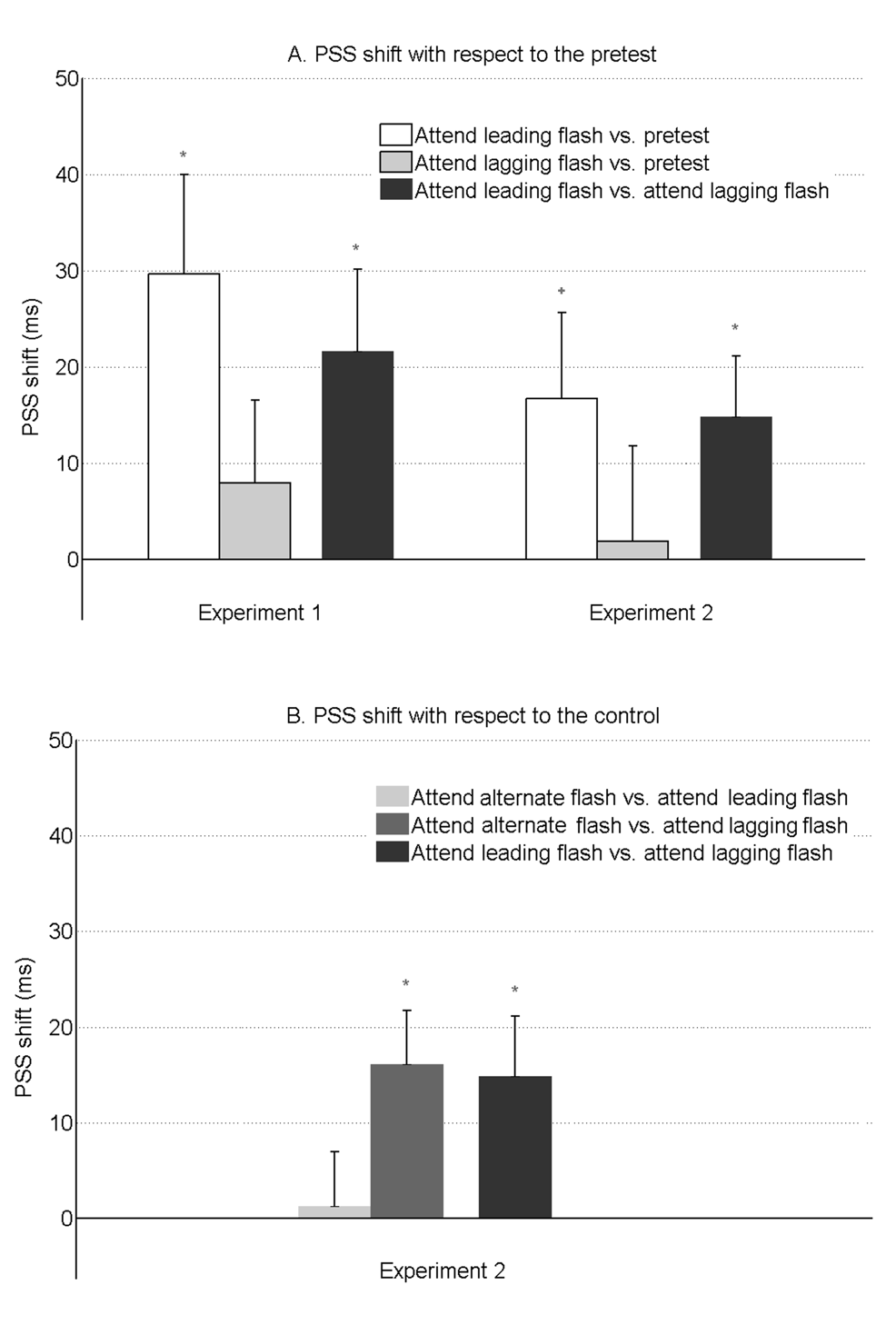

Supplement: Figure S3 — A. PSS shift differences between conditions with respect to the pretest in Experiment 1 and Experiment 2. PSS shifts were obtained from the difference between mean PSS values for the attend leading and attend lagging flash condition and each attended condition with respect to the pretest. B. PSS shift differences between conditions with respect to attend alternate condition in Experiment 2. PSS shifts were obtained from the difference between mean PSS values for the attend leading and attend lagging flash condition and each of the attended condition with respect to a baseline equated for task demands (attend alternate). Positive PSS values indicate that the tested flash had to be presented before the tone to perceive audiovisual events simultaneous. The asterisks denote significant differences of the PSS, the cross denotes a marginally significant difference (p = 0.078) and the error bars denote the SEM calculated from the difference between conditions. (TIF) [file pone.0099311.s003.tif]

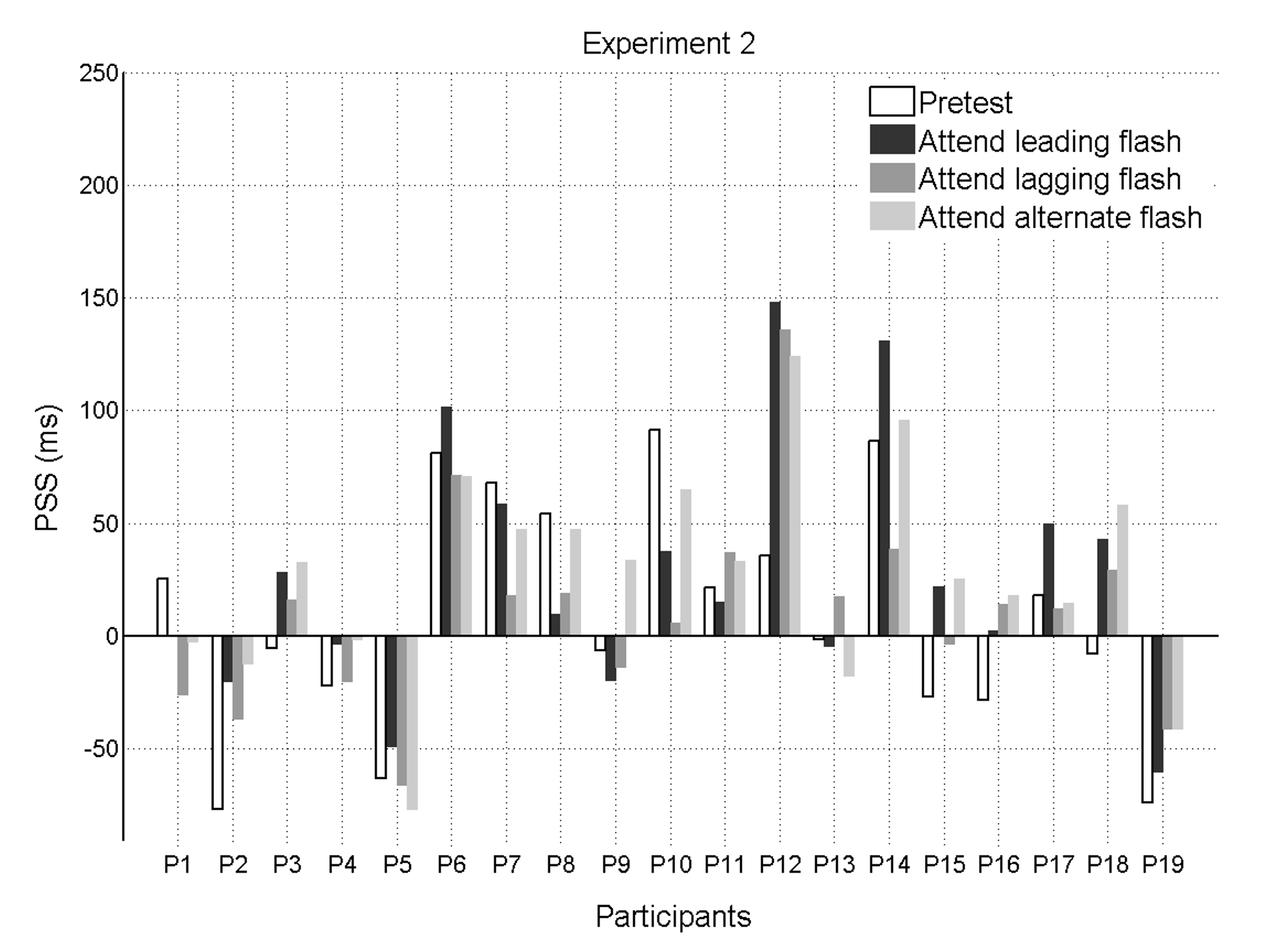

Supplement: Figure S4 — Individual PSS mean values for each condition in Experiment 2 . (TIF) [file pone.0099311.s004.tif]
