# Supplementary material for: Selective Attention Modulates the Direction of Audio-Visual Temporal Recalibration
Source: PLoS One. 2014 Jul 8;9(7):e99311. doi: 10.1371/journal.pone.0099311 (PMC4086723; doi:10.1371/journal.pone.0099311)
Supplement: Table S1 — PSS, Sigma and R2 mean estimates for each condition in Experiment 1 and 2 . (DOC) [file pone.0099311.s005.doc]

**Table S1. PSS, Sigma and R² mean estimates for each condition in Experiment 1 and 2.**

|  | **conditions** | **PSS ± SEM** | **sigma ± SEM** | **R² ± SEM** |
| --- | --- | --- | --- | --- |
| **Experiment 1** (n=14) | Pretest | 11.31 ± 15.89 | 0.09 ± 0.01 | 0.78 ± 0.02 |
|  | Attend leading flash | 40.93 ± 17.83 | 0.09 ± 0.01 | 0.75 ± 0.03 |
|  | Attend lagging flash | 19.3 ± 14.41 | 0.09 ± 0.01 | 0.74 ± 0.03 |
| **Experiment 2** (n=19) | Pretest | 8.96 ± 11.93 | 0.10 ± 0.01 | 0.73 ± 0.02 |
|  | Attend leading flash | 25.67 ± 12.63 | 0.10 ± 0.01 | 0.71 ± 0.02 |
|  | Attend lagging flash | 10.48 ± 10.2 | 0.10 ± 0.01 | 0.73 ± 0.03 |
|  | Attend alternate flash | 26.93 ± 10.91 | 0.10 ± 0.01 | 0.71 ± 0.02 |

Number of subjects included in each analysis is reported (n).
