# Supplementary material for: Selective Attention Modulates the Direction of Audio-Visual Temporal Recalibration
Source: PLoS One. 2014 Jul 8;9(7):e99311. doi: 10.1371/journal.pone.0099311 (PMC4086723; doi:10.1371/journal.pone.0099311)
Supplement: Table S2 — Reported Wilcoxon signed Rank test values for related pair samples conditions: PSS in Experiment 1 and 2 . (DOC) [file pone.0099311.s006.doc]

**Table S2. Reported Wilcoxon signed Rank test values for related pair samples conditions: PSS in Experiment 1 and 2.**

|  | **Conditions** | **z (Wilcoxon test)** | **p (significant values)** |
| --- | --- | --- | --- |
| **Experiment 1** (n=14) | Attend leading vs. lagging flash | z=-2.66 | p=0.03 |
|  | Pretest vs. attend leading flash | z=-2.417 | p=0.016 |
|  | Pretest vs. attend lagging flash | z=-0.847 | p=0.397 |
| **Experiment 2** (n=19) | Attend leading vs. lagging flash | z=-2.173 | p=0.03 |
|  | Attend alternate vs. attend leading flash | z=-0.241 | p=0.809 |
|  | Attend alternate vs. attend lagging flash | z=-2.374 | p=0.018 |
|  | Pretest vs. attend leading flash | z=-1.771 | p=0.077 |
|  | Pretest vs. attend lagging flash | z=-0.161 | p=0.872 |

Number of subjects included in each analysis is reported (n).
