# Supplementary material for: Selective Attention Modulates the Direction of Audio-Visual Temporal Recalibration
Source: PLoS One. 2014 Jul 8;9(7):e99311. doi: 10.1371/journal.pone.0099311 (PMC4086723; doi:10.1371/journal.pone.0099311)
Supplement: Table S3 — Mean proportion of oddball detection for each condition in Experiment 1 and 2 . (DOC) [file pone.0099311.s007.doc]

**Table S3. Mean proportion of oddball detection for each condition in Experiment 1 and 2.**

|  | **Conditions** | **Oddball detection rate ± SEM** |
| --- | --- | --- |
| **Experiment 1** (n=14) | Attend leading flash | 0.59 ± 0.08 |
|  | Attend lagging flash | 0.64 ± 0.08 |
| **Experiment 2** (n=19) | Attend leading flash | 0.53 ± 0.07 |
|  | Attend lagging flash | 0.66 ± 0.05 |
|  | Attend alternate flash | 0.56 ± 0.06 |

Number of subjects included in each analysis is reported (n).
