# Supplementary material for: Selective Attention Modulates the Direction of Audio-Visual Temporal Recalibration
Source: PLoS One. 2014 Jul 8;9(7):e99311. doi: 10.1371/journal.pone.0099311 (PMC4086723; doi:10.1371/journal.pone.0099311)
Supplement: Table S4 — Reported Wilcoxon signed Rank test values for related pair samples conditions: Proportion of oddball detection in Experiment 1 and 2 . (DOC) [file pone.0099311.s008.doc]

|  | **Conditions** | **z (Wilcoxon test)** | **p (significant values)** |
| --- | --- | --- | --- |
| **Experiment 1** (n=14) | Attend leading flash vs. attend lagging flash | z=-1.083 | p=0.279 |
| **Experiment 2** (n=19) | Attend leading flash vs. attend lagging flash | z=-1.938 | p=0.053 |
|  | Attend leading flash vs. attend alternate flash | z=-0.327 | p=0.744 |
|  | Attend lagging flash vs. attend alternate flash | z=-1.254 | p=0.210 |

**Table S4. Reported Wilcoxon signed Rank test values for related pair samples conditions:** Proportion of oddball detection **in Experiment 1 and 2.**

Number of subjects included in each analysis is reported (n).
