# Supplementary material for: Selective Attention Modulates the Direction of Audio-Visual Temporal Recalibration
Source: PLoS One. 2014 Jul 8;9(7):e99311. doi: 10.1371/journal.pone.0099311 (PMC4086723; doi:10.1371/journal.pone.0099311)
Supplement: Table S5 — Proportion of oddball detection for each participant in Experiment 1 and 2 . (DOC) [file pone.0099311.s009.doc]

**Table S5. Proportion of oddball detection for each participant in** Experiment 1 and 2.

| **Experiment 1: Participants** | **Attend leading flash** | **Attend lagging flash** |
| --- | --- | --- |
| 1 | 0.43 | 0.23 |
| 2 | 0.67 | 0.90 |
| 3 | 0.17 | 0.60 |
| 4 | 0.88 | 0.90 |
| 5 | 0.58 | 0.78 |
| 6 | 0.75 | 1 |
| 7 | 1 | 1 |
| 8 | 0.94 | 1 |
| 9 | 0.80 | 0.68 |
| 10 | 0.40 | 0.60 |
| 11 | 0.25 | 0.27 |
| 12 | 0.19 | 0.48 |
| 13 | 0.90 | 0.38 |
| 14 | 0.30 | 0.21 |

Proportion of oddball detection for each participant/condition in Experiment 1.

| **Experiment 2: Participants** | **Attend leading flash** | **Attend lagging flash** | **Attend alternate flash** |
| --- | --- | --- | --- |
| 1 | 0.40 | 0.50 | 0.33 |
| 2 | 0.34 | 0.39 | 0.76 |
| 3 | 1 | 0.60 | 0.37 |
| 4 | 0.88 | 0.60 | 0.69 |
| 5 | 0.06 | 0.54 | 0.63 |
| 6 | 0.56 | 0.77 | 0.80 |
| 7 | 0.17 | 0.75 | 0.51 |
| 8 | 0.90 | 0.92 | 0.83 |
| 9 | 0.70 | 0.69 | 0.29 |
| 10 | 0.88 | 0.39 | 0.65 |
| 11 | 0.65 | 1 | 0.23 |
| 12 | 0.07 | 0.75 | 0.33 |
| 13 | 0.44 | 0.46 | 0.75 |
| 14 | 0.29 | 1 | 1 |
| 15 | 1 | 1 | 1 |
| 16 | 0.52 | 0.53 | 0.43 |
| 17 | 0.10 | 0.30 | 0.49 |
| 18 | 0.60 | 0.70 | 0.20 |
| 19 | 0.54 | 0.76 | 0.41 |

Proportion of oddball detection for each participant/condition in Experiment 2.
