# Supplementary material for: Selective Attention Modulates the Direction of Audio-Visual Temporal Recalibration
Source: PLoS One. 2014 Jul 8;9(7):e99311. doi: 10.1371/journal.pone.0099311 (PMC4086723; doi:10.1371/journal.pone.0099311)
Supplement: Supporting Information S1 — (DOC) [file pone.0099311.s010.doc]

**Supporting Information**

**Temporal bisection task.** An adaptation trial consisting of a flash-tone-flash sequence was presented at the centre of a black square pedestal with the same background characteristics and stimuli properties of the experiment. A fixation marker was presented and the session started with the tone delivered at the physical middle between both flashes (separated by 235 ms from each flash). The flashes were isoluminant and colored green and red (order counterbalanced across participants). Subjects judged which of the two flashes appeared to be temporally closer to the tone in an adaptive procedure (20 ms steps) by pressing two different keys (for example, see Burr, Banks and Morrone (2009). Auditory dominance over vision in the perception of interval duration. *Experimental Brain Research*, **198**, Issue 1, pp 49-57 (doi: 10.1007/s00221-009-1933-z) for similar audiovisual temporal bisection tasks). Participants also had the possibility to repeat the trial by pressing a third key. Once the tone was reported as bisecting the interval between the two flashes, participants were asked to press the space bar. The chosen flash-tone-flash asynchrony was presented to the participant for 3 times. They performed this task at least 2 times, until their values remained stable. During the bisection task, explicit instructions about attention were not given.

**Flicker fusion task.** The green and grey colour was adjusted for isoluminance with the red one, using a colour flicker fusion task adapted from Cavanagh, MacLeod, and Antis (1987). Equiluminance: spatial and temporal factors and the contribution of blue-sensitive cones. *Journal of the Optical Society of America. A, Optics and image science* **4**, 1428–38. Participants attended to a ring flickering between green and red every 11.8 ms or between grey and red in a subsequent trial, at the centre of a black square pedestal with the same background and stimulus characteristics of the experimental task. After exploring the stimulus, they were instructed to minimize the flickering by pushing up or down arrows. The maximum and minimum level of luminance was adjusted for each of the colour pairs. After each isoluminance match pairing, an example of the coloured rings was presented to the participants. The experimenter remained inside the testing room during this procedure in order to ensure participants understood well the task.

Addendum

To achieve isoluminance, the green and grey luminance and contrasts were adjusted for each participant relative to a fixed red background luminance of 14.66 cd/m² and a fixed red contrast set at 0.34. The background luminance of the green colour was adjusted overall in Exp. 1 and Exp. 2 around 13%. That is, the mean green background luminance was 16.32 cd/m² (SEM=0.74) and 16.65 cd/m² (SEM=0.67) for Exp.1 and 2 respectively. The mean contrast for the green was 0.32 (SEM=0.01) and 0.31 (SEM=0.031) for Exp.1 and 2 respectively. Thus, the green contrast was reduced respect to the red around 6.6%. On the other hand, the grey luminance values were adjusted around 16%. Mean grey background was 16.9 cd/m² (SEM=0.67) and 16.7 cd/m² (SEM=0.49) for Exp.1 and 2 respectively. The mean contrast for the grey colour was 0.29 (SEM=0.01) and 0.31 (SEM=0.01) for Exp.1 and 2, respectively. Thus, the grey contrast was reduced around 12.3% respect to the red one.
